# Removal of Arsenate and Arsenite in Equimolar Ferrous and Ferric Sulfate Solutions through Mineral Coprecipitation: Formation of Sulfate Green Rust, Goethite, and Lepidocrocite

**DOI:** 10.3390/soilsystems4040068

**Published:** 2020-11-23

**Authors:** Chunming Su, Richard T. Wilkin

**Affiliations:** Groundwater Characterization and Remediation Division, Center for Environmental Solutions and Emergency Response, Office of Research and Development, United States Environmental Protection Agency, 919 Kerr Research Drive, Ada, OK 74820, USA;

**Keywords:** arsenic coprecipitation, redox transformation, iron oxides, X-ray absorption spectroscopy, Raman spectroscopy

## Abstract

An improved understanding of in situ mineralization in the presence of dissolved arsenic and both ferrous and ferric iron is necessary because it is an important geochemical process in the fate and transformation of arsenic and iron in groundwater systems. This work aimed at evaluating mineral phases that could form and the related transformation of arsenic species during coprecipitation. We conducted batch tests to precipitate ferrous (133 mM) and ferric (133 mM) ions in sulfate (533 mM) solutions spiked with As (0–100 mM As(V) or As(III)) and titrated with solid NaOH (400 mM). Goethite and lepidocrocite were formed at 0.5–5 mM As(V) or As(III). Only lepidocrocite formed at 10 mM As(III). Only goethite formed in the absence of added As(V) or As(III). Iron (II, III) hydroxysulfate green rust (sulfate green rust or SGR) was formed at 50 mM As(III) at an equilibrium pH of 6.34. X-ray analysis indicated that amorphous solid products were formed at 10–100 mM As(V) or 100 mM As(III). The batch tests showed that As removal ranged from 98.65–100%. Total arsenic concentrations in the formed solid phases increased with the initial solution arsenic concentrations ranging from 1.85–20.7 g kg^−1^. Substantial oxidation of initially added As(III) to As(V) occurred, whereas As(V) reduction did not occur. This study demonstrates that concentrations and species of arsenic in the parent solution influence the mineralogy of coprecipitated solid phases, which in turn affects As redox transformations.

## 1. Introduction

Arsenic (As) adsorption onto, desorption from, and coprecipitation with aquifer materials, particularly iron oxide minerals and clays are key processes that control As fate and mobility in the subsurface [1,2]. Additionally, redox reactions involving biogeochemical cycling of macro elements of iron, sulfur, and carbon also play an important role [1,2]. In oxic aquifers under alkaline conditions such as the Central Oklahoma aquifer, dissolution of carbonate rocks such as dolomite leads to an increase in pH, which promotes desorption of anions from solid geomaterials leading to elevated levels of dissolved arsenic [3] mostly as arsenate. In anoxic aquifers such as those in Bangladesh, high levels of dissolved As are believed to be related to partial or complete reductive dissolution of iron oxides driven by microbial decomposition of organic matter [4]. Inorganic arsenic species are common contaminants in arsenic-rich groundwaters and organic forms such as methylated species are typically observed at low or negligible concentrations [1]. Sorption of arsenic on natural organic matter (NOM) is of minor or negligible importance except with NOM-metal complexes that may strongly bind As(V) and As(III) anions through metal bridging mechanisms [5].

Based on thermodynamics, arsenate (As(V) or H_n_AsO_4_^n−3^, n = 0–3) is expected to be the predominant inorganic arsenic species in groundwater under high pH and oxidizing conditions; whereas, arsenite (As(III) or H_n_AsO_3_^n−3^, n = 0–3) should be dominant under low pH and reducing conditions [6]. However, redox transformations of As(V) to As(III) and vice versa are kinetically limited so that non-equilibrium distribution of As(V) and As(III) is often observed [7]. Reductive dissolution of iron oxides has been proposed as a leading mechanism for high levels of dissolved As in reducing groundwaters, but no clear correlation between dissolved Fe and As in groundwater has been found. Total dissolved Fe concentrations are generally much lower than those that would be supported by complete dissolution of iron(III) oxides. This could be explained by reoxidation of Fe(II) [8] and precipitation of Fe(II) solid phases such as ferrous sulfide (FeS), pyrite (FeS_2_), siderite (FeCO_3_), vivianite (Fe(II)_3_(PO_4_)_2_·8H_2_O), symplesite (Fe(II)_3_(As(V)O_4_)_2_·8H_2_O), and green rusts (GRs) [9–11].

GRs are Fe(II)-Fe(III) layered double hydroxides that consist of Fe(II)_(1−x)_Fe(III)_x_(OH)_2_^x+^ brucite-like cation layers and interlayers, [(x/n)A^n−^·(mx/n)H_2_O]^x−^ that contain A^n−^ anions and mx/n water molecules to balance the cation layer charge [12].The typical Fe(II)/Fe(III) molar ratios in GR phases are 2 and 3, but a range of Fe(II)/Fe(III) ratios from ≥1 to 3.0 have been reported [13–18]. Synthesized GRs chemically reduce nitrate to ammonium ions [19], Cr(VI) to Cr(III) [20,21]; U(VI) to UO_2_ [22]; and Ag(I), Au(III), Cu(II), Hg(II) to Ag(0), Au(0), Cu(0), and Hg(0) [23], respectively. Reductive dechlorination of CCl_4_ by GRs has also been reported [24–26]. GRs are initial corrosion products of zerovalent iron that is used in permeable reactive barrier technologies to remove groundwater arsenic [27]. GRs are reportedly found in oxidation to reduction transition zones in soils and sediments, although clear X-ray diffraction (XRD) evidence is not common [28,29]. Direct XRD evidence, however, was demonstrated for the presence of carbonate green rust (CGR) in a groundwater sample taken below the water table from fractures in granite [30]. Two common GRs are: 1) iron(II, III) hydroxycarbonate green rust (CGR, Fe(II)_4_Fe(III)_2_(OH)_12_CO_3_, formula from Hansen, 1989 [31]; Fe(II)_4_Fe(III)_2_(OH)_12_CO_3_·3H_2_O, formula from Génin et al., 2005 [32]; Fe(II)_4_Fe(III)_2_(OH)_12_CO_3_·2H_2_O, formula from Drissi et al., 1995 [33]); and 2) iron(II, III) hydroxysulfate green rust (sulfate green rust or SGR, Fe(II)_4_Fe(III)_2_(OH)_12_SO_4_·8H_2_O, formula from Simon et al., 2003 [34]; Fe(II)_4_Fe(III)_2_(OH)_12_SO_4_·3H_2_O, formula from Randall et al., 2001 [35]. GRs may be involved in the speciation and redox processes of oxyanionic arsenic species, as our previous study has shown that As(III) is partially oxidized by preformed CGR under anoxic conditions [36]. Oxidation of arsenic species is beneficial and desirable because As(V) is known to be less toxic than As(III), and the sorbed form of As(V) is therefore a preferred species over As(III). GRs have a high capacity to adsorb both As(V) and As(III) presumably at the edge sites of GRs where singly coordinated OH groups reside [11]. The outer surfaces of GR particles are dominated by OH groups that are not highly reactive sites due to their coordination to three iron atoms in the trioctahedral Fe(II)-Fe(III) metal hydroxide layers. Singly coordinated OH groups are the most reactive sites, e.g., with respect to phosphate sorption [37]. Coprecipitation of As(V) with hydrous ferric oxide and its subsequent transformation to more crystalline phases (hematite and goethite) is reportedly related to arsenate solid loading [38].

X-ray diffraction is widely used for mineral identification and Raman spectroscopy has been used to augment mineral identification and characterization [39]. The X-ray absorption near edge spectroscopy (XANES) approach can determine oxidation states of arsenic by resolving small shifts in the energy position of the As K absorption edge [40]. Little is known about how the coprecipitates formed in situ with As(V) or As(III) in the presence of both Fe(II) and Fe(III) affect the arsenic solubility and transformation. Based on the shape and energy edge position, XANES spectra can be used to identify sensitive changes in the formal valence state and coordination environment of samples. This laboratory study focuses on the solution conditions dominated by sulfate, which simulates acid mine drainage or sulfate dominated groundwater systems. The objectives were therefore to examine: (1) the influence of As(V) and As(III) concentrations on the formation of green rust and iron oxides; (2) the extent of arsenic removal by coprecipitation with solid phases; and (3) the extent of redox transformation of arsenic species during coprecipitation. This study did not intend to elucidate detailed mechanisms of As transformation due to its complexity; rather it aimed at evaluating if and to what extent the presence of As(V) and As(III) stabilize green rust in the competitive formation of various iron oxides.

## 2. Materials and Methods

### 2.1. Chemicals

All chemicals used were analytical reagent grade without further purification. Degassed and deionized water (ultra high purity N_2_ gas bubbling for 30 min), disodium hydrogen arsenate heptahydrate, Na_2_HAsO_4_·7H_2_O (Baker, Phillipsburg, NJ, USA), and sodium arsenite, NaAsO_2_ (Baker, Phillipsburg, NJ, USA) were used to prepare stock solutions (0.01335 M and 0.1000 M As(V) or As(III)).

### 2.2. Formation of Coprecipitation Solids

The ratio of dissolved Fe(II)/Fe(III) varies depending on redox potential in natural water with Fe(II) being dominant in groundwater and Fe(III) dominant in aerated acid mine drainage. For simplicity, a 1:1 ratio was used in this study. Added to each of the 50-mL centrifuge tubes (actual volume = 41.64 ± 0.17 mL, n = 10) were 0.928 g of FeSO_4_·7H_2_O (ferrous sulfate heptahydrate, Sigma-Aldrich). This would result in a final concentration of 133 mM Fe(II) for a total volume of 25 mL solution. Next, 0.815 g of Fe_2_(SO_4_)_3_·5H_2_O (ferric sulfate pentahydrate, Baker, Phillipsburg, NJ, USA) were added into each of the centrifuge tubes. This would result in a final concentration of 133 mM Fe(III). Thus, the molar ratio of Fe(II):Fe(III) was 1:1. Appropriate amounts of degassed and deionized water and arsenic stock solution (with a total solution volume equal to 25 mL) were added and the centrifuge tubes were agitated manually to dissolve all solids. The resulting As(V) or As(III) concentrations were 0, 0.5, 1.0, 5.0, 10, 50, and 100 mM. Next, 0.40 g of NaOH granules (sodium hydroxide, Sigma, St Louis, MO, USA) were added into each tube, followed by adding 0.710 g of Na_2_SO_4_ (sodium sulfate anhydrous, Mallinckrodt, St Louis, MO, USA). The final concentration of Na_2_SO_4_ was 0.2 M (total [SO_4_^2−^]_0_ = 0.533 M including ferric and ferrous sulfate salts), and the final concentration of NaOH was 0.4 M. Thus, the molar ratio of [OH^−^]:[total Fe] was 1.5:1. The centrifuge tubes were hand-shaken for 5 min and then were covered with aluminum foil and the resulting precipitates were aged for 24 h inside an anaerobic glovebox (3–5% H_2_ in N_2_). The headspace of the centrifuge tube was filled with about 16.64 mL of air of which 3.49 mL was O_2_ (21% of air) so that the tube contained 0.1561 millimoles of O_2_ as compared to 3.325 millimoles of Fe(II). Thus the potential of Fe(II) oxidation and As(III) oxidation by dissolved O_2_ during the 5-min reaction was limited because the other oxidant Fe(III) was dominant at 3.325 millimoles. Assuming all of the O_2_ was consumed to oxidize Fe(II), only less than 4.69% of added Fe(II) could be oxidized.

### 2.3. X-Ray Diffraction (XRD) Analysis

After 24 h of equilibration of mineral suspensions, the tubes were shaken well manually, and 1.0 mL of each suspension was pipetted out into a syringe connected to a filter disk and filtered through a 0.22-μm Millipore nitrocellulose membrane. The solids on the membrane were rinsed with 10 mL of deionized and degassed water to remove soluble salts before being dried for 2 h on the filter disk inside the anaerobic glovebox (relative humidity = 40–60% without adjustment; constant circulation was maintained using an electrical fan under an O_2_ removing catalyst). A portion of the dried solid (about 50 mg) was mixed with a drop of glycerol (to prevent oxidation by air during XRD analysis) on a zero-background quartz slide. The slide was taken out of the glovebox and scanned with a Rigaku Miniflex X-ray diffractometer (Rigaku Corp., The Woodlands, TX, USA) at a scan speed of 0.5° 2θ min^−1^ from 5 to 95° 2θ (Fe Kα_2_ radiation, λ = 1.94 Å, 30 keV and 15 mA). NIST 640b standard reference material (silicon powder) was periodically scanned as a quality control check of d-spacing accuracy. Peak analysis was performed with Jade software and sample XRD patterns were compared with those of the International Center for Diffraction Data (ICDD)’s Powder Diffraction File (PDF) database.

### 2.4. X-ray Absorption Spectroscopy Analysis

Arsenic XANES was used to determine the arsenic oxidation state for the coprecipitates formed at different initial As(III) concentrations (0.5, 1.0, 5.0, 10, 50, and 100 mM). Coprecipitated samples for the XANES measurements were homogenized and ground to a fine powder, loaded into Teflon sample holders and then sealed with Kapton tape in a N_2_-filled glove box to avoid oxidation at room temperature. Prepared samples were transported and kept without exposure to air prior to XANES data collection. Arsenic K-edge (11.867 keV) XANES were collected using the MR-CAT beamline 10-ID at the Advanced Photon Source (APS) at Argonne National Laboratory (ANL, Argonne, IL, USA). The APS electron storage ring operated at 7 GeV with a top-up fill status. All spectra were collected in transmission mode with a Si(111) monochromator crystal. XANES analyses were conducted by scanning across the absorption edge region in three segments with a 2- to 3-s dwell time by increment. At each absorption edge, 2–3 successive scans were collected and averaged. Energy calibration of samples used sodium arsenate from Aldrich which has a well-defined oxidation state for As XANES (11874 eV edge position). The arsenic K-edge of sodium arsenate was run simultaneously with samples to check for potential energy shifts during the run as well as possible As(III) oxidation during the data collection. Extraction of normalized XANES spectra from the raw data was performed using the software Athena [41].

### 2.5. Raman Spectroscopy Analysis

Samples of coprecipitated solids were transferred onto a clean sample plate and packed appropriately for the convenience of focus under Raman microscopy (SENTERRA, Bruker Optics, MA, USA). The Raman spectroscopy analysis was conducted at a wavelength of 532 nm. The instrument calibration was checked using a silicon wafer at a Raman shift of 520.5 cm^−1^.

### 2.6. Aqueous Solution Analysis

The centrifuge tubes containing the remaining mineral suspensions were taken out of the glovebox and centrifuged at 3600*g* × rpm (relative centrifugal force = 2600*g* ×) for 15 min and then transferred back to the anaerobic glovebox. Oxidation during centrifugation was negligible because the tubes were tightly capped and centrifugation time was short. The supernatant solutions were measured for pH using a combination pH electrode and for redox potential using a Pt redox electrode. The redox electrode was checked with a ferrocyanide buffer solution. The redox potential values were corrected to Eh values relative to standard hydrogen potential. The supernatant solutions were filtered through 0.22-μm Millipore nitrocellulose membranes into 30-mL amber plastic bottles and acidified to pH less than 2 by adding 0.1 mL of 5 M HCl. Dissolved ferrous iron was measured by the 1,10-phenanthroline colorimetric method using a Hach spectrophotometer inside the anaerobic glovebox. Total dissolved Fe and As in the filtered solution were determined with Inductively Coupled Plasma-Optical Emission Spectrometry (ICP-OES, Perkin Elmer Optima 8300 DV, Waltham, MA, USA). Solution arsenic speciation analysis was performed with Liquid Chromatography-Inductively Coupled Plasma-Mass Spectrometry (LC-ICP-MS, Thermo Electron Spectra HPLC with Thermo Electron X series II ICP-MS, Thermo Fisher Scientific, Waltham, MA, USA). The LC system separates the species via a Hamilton PRP-X100 anion exchange column and elution using a mobile phase of 10 mM (NH_4_)H_2_PO_4_/NH_4_NO_3_ and is then pumped to the ICP-MS system, which quantifies the elemental species. Calibration of ICP-MS was carried out using 0, 100, 250, 500, 1000, and 2000 μg L^−1^ for As and the detection limits were 17 μg L^−1^ for As(V) and 10 μg L^−1^ for As(III). The spike percent recovery was within 100 ± 10%.

### 2.7. Arsenic Concentration in Solid Phases

The remaining solids in centrifuge tubes from the in situ coprecipitation experiments were mixed with 30 mL of degassed and deionized water and equilibrated for four days with manual shaking inside the anaerobic glovebox. The tubes were then taken out and centrifuged at 3600*g* × rpm for 30 min. The tubes were moved back to the glovebox and the supernatant solutions were removed. The caps on the tubes were removed so that the washed solids were dried in the glovebox under a humidity of 40–60% for 60 days, at which time solids were visually dried. Duplicates of 50 mg of the dried solids were mixed with 5 mL of 6.0 M HCl in a 50-mL centrifuge tube with the green rust completely dissolving after 30 min and the iron oxides completely dissolving after 24 h. Twenty-five milliliters of degassed deionized water were added to the digested samples before being analyzed for total dissolved As and Fe using ICP-OES with a method detection limit of 0.2 mg L^−1^ for As and 0.05 mg L^−1^ for Fe.

## 3. Results

### 3.1. Iron and Arsenic Removal

Significant amounts of residual dissolved ferrous iron remained 24 h after coprecipitation (21.9 to 91.2 mM versus [Fe^2+^]_0_ = 133 mM in the As(V) system, Table 1; 47.9 to 92.2 mM versus [Fe^2+^]_0_ = 133 mM in the As(III) system, Table 2). These dissolved Fe(II) concentrations were roughly comparable to the total dissolved iron. Iron removal ranged from 64% to 90% in the As(V) systems and 63 to 79% in the As(III) systems. The incomplete iron removal is related to the acidic final pH of the suspensions; the suspension pH ranged from 3.73 to 6.62 and indicated a lack of sufficient hydroxyl ions to precipitate dissolved Fe(II) completely out of solution. Increasing the concentrations of added As(V) or As(III) generally led to lower residual dissolved Fe(II) in the aqueous solution after coprecipitation (Tables 1 and 2). The final Eh values ranged from 31 to 227 mV in the As(V) systems and from 18 to 417 mV in the As(III) systems (Tables 1 and 2), confirming moderately reducing conditions in the mineral suspensions. As(V) removal ranged from 99.95–100% in the As(V) system (Table 1) and 98.65–99.68% in the As(III) system (Table 2).

### 3.2. Mineralogy of Coprecipitated Solids

**Figure 1.**
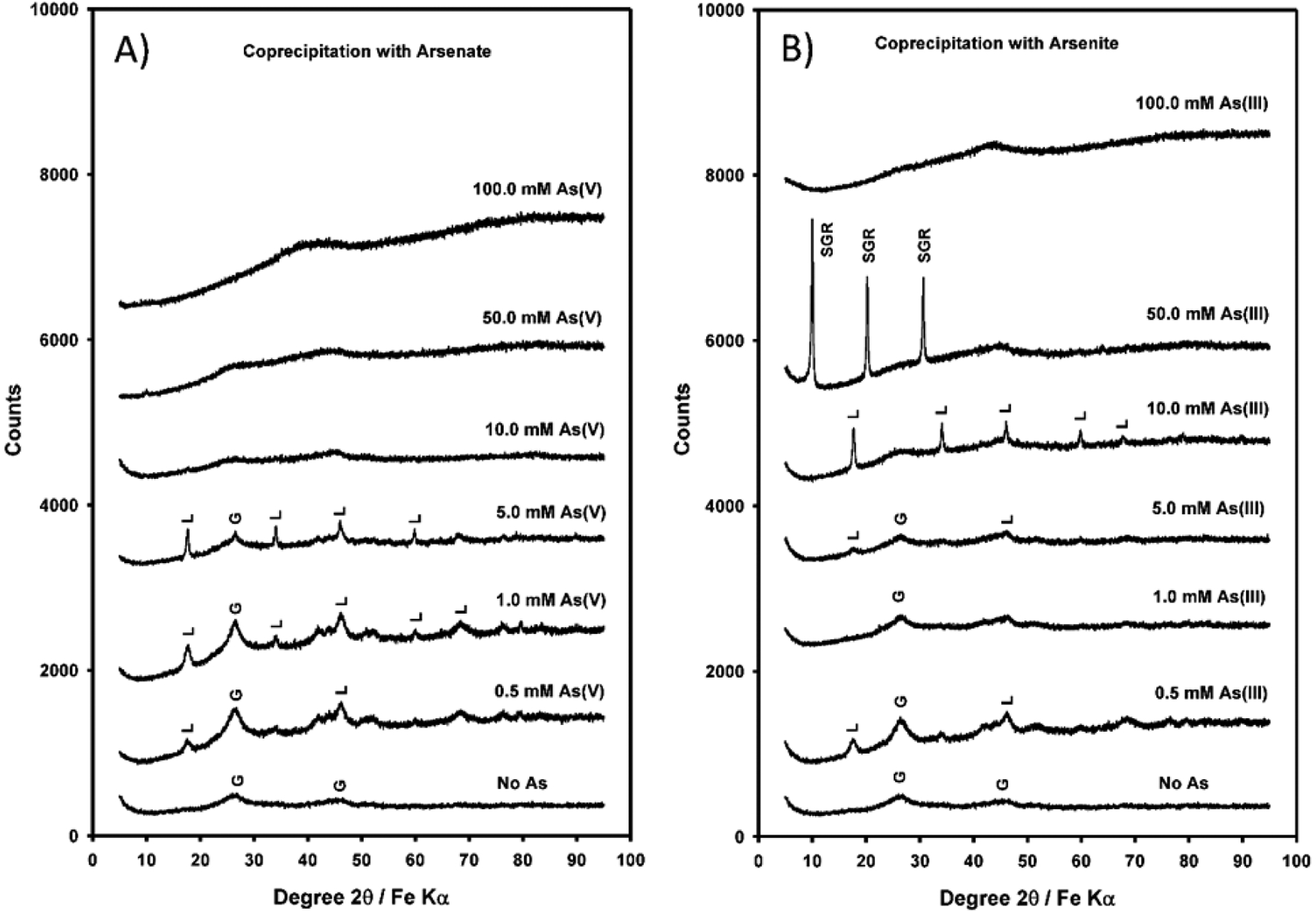
(**A**): X-ray diffractogram of coprecipitate minerals formed at varying dissolved arsenate concentrations of 0, 0.5, 1.0, 5.0, 10.0, 50.0, and 100 mM (L = lepidocrocite, G = goethite); (**B**) X-ray diffractogram of coprecipitate minerals formed at varying dissolved arsenite concentrations of 0, 0.5, 1.0, 5.0, 10.0, 50.0, and 100 mM (SGR = sulfate green rust, L = lepidocrocite, G = goethite).

In the As(V) systems, mineralogy of formed solid phases was dependent on the concentrations of added As(V) (Figure 1). Lepidocrocite (γ-FeOOH, PDF 08–0098) and poorly crystalline goethite (α-FeOOH, PDF 29–0713) were the crystalline phases identified at initial concentrations of As(V) from 0 to 5 mM with the final pH values ranging from 4.09 to 6.59, and Eh values ranging from 31 to 366 mV (Figure 1, Table 1). Lepidocrocite and goethite did not show any magnetism as tested using a hand-held magnet bar. The presence of ferrihydrite (PDF 29–0712) cannot be completely ruled out because it is close to being X-ray amorphous. No SGR (PDF 13–0092) was formed in any of the sulfate systems when As(V) was added. Lepidocrocite formation does not require ferrous ion, therefore large portions of added ferrous ions resided in the aqueous phase (Tables 1 and 2) and some ferrous ions were expected to be adsorbed on the surface of lepidocrocite particles.

(1) $${\text{Fe}}^{3+}\;+\;2{\text{H}}_{2}\text{O}\;=\;\gamma -\text{FeOOH}\;+\;3{\text{H}}^{+}$$

Goethite precipitated at the expense of lepidocrocite in the absence of chloride in this study, consistent with an earlier study that showed the presence of chloride favors the formation of lepidocrocite and the presence of sulfate favors the formation of goethite [42]. No magnetite was identified in the present study, in agreement with previous reports that showed magnetite only formed at alkaline pH during the abiotic oxidation of Fe(II) in the presence of As(V) [43] or during simultaneous oxidation of As(III) and Fe(II) [44]. Another earlier study showed that magnetite was formed after initially precipitated lepidocrocite and green rust were transformed to magnetite [45]. That study used much lower concentrations of dissolved Fe(II) and Fe(III) (Fe(II)/Fe(III) = 0.5), and As(V) and As(III) in the coprecipitation experiment. No crystalline ferrous arsenite solid such as H_7_Fe_4_(AsO_3_)_5_ was identified in this study, although this phase was reportedly formed when millimolar concentrations of dissolved Fe(II) and As(III) were mixed at pH < 7.5 [46]. The presence of dissolved Fe(III) may have inhibited the formation of solid ferrous arsenite in our study.

No GRs formed in the As(V) systems (Figure 1). GRs are known to readily transform. A recent study showed that SGR formed at pH 9 and Fe(II):Fe(III) ratios of 0.5:1 and 1:1 was transformed to magnetite under O_2_-free conditions, whereas at pH 7 and Fe(II):Fe(III) ratios of 0.5:1, SGR was stable, with no transformation to magnetite [47]. SGR may also transform to magnetite and Fe(OH)_2_ in the presence of excess OH^−^ ions [48]. In the present test, the final pH values ranged from 4.83 to 6.59 such that the lack of SGRs was not entirely due to insufficient OH^−^ ions. Rather, the presence of As(V) seemed to prohibit the formation of SGRs.

An earlier study showed that SGR formed in solutions of FeSO_4_ neutralized by NaOH and aerated with air transformed completely to lepidocrocite within two hours of SGR formation [49]. Another study showed that phosphate ions favor lepidocrocite over goethite formation when initially formed green rust (most likely CGR) was oxidized by bubbling air because lepidocrocite has a layered structure (like its precursor green rust) and a structure less dense than that of goethite, thereby requiring less complete removal of the green rust interlayer phosphate to form [50]. That study demonstrated that goethite dominates at low phosphate concentrations (P:Fe < 0.005); whereas, lepidocrocite dominates at high phosphate concentrations (P:Fe = 0.01:1 to 0.2:1). As expected, our study showed a similar effect of As(V) on the mineralogy of coprecipitates because arsenate and phosphate are structurally and chemically similar. No scorodite [Fe(III)As(V)O_4_·2H_2_O] was found in the systems we studied here; this may not be surprising because scorodite only forms under strongly acidic conditions [51–53]. Poorly crystalline ferric arsenate that resembles its scorodite precursor was reported to form from equimolar Fe(III)-As(V) solutions of sulfate or nitrate in the pH range 2 to 8 [54]. Ferrihydrite or schwertmannite (Fe_8_O_8_(OH)_6_SO_4_)-like solids were shown to form when Fe(III) (3–236 mM) as Fe_2_(SO_4_)_3_ was reacted with As(V) (0.24–18 mM) at pH 3.5–7.0 [55]. In our study, amorphous materials containing varying amounts of As(V) (4.1 to 17.1%, Table 1) were formed at initial 10 to 100 mM As(V). The molar ratio of Fe(III):As(V) in these precipitates are variable and unequal to 1:1, thus, scorodite-like amorphous ferric arsenate is unlikely to be present. Rather, amorphous HFO with coprecipitated As(V) is more likely to have been formed.

**Figure 2.**
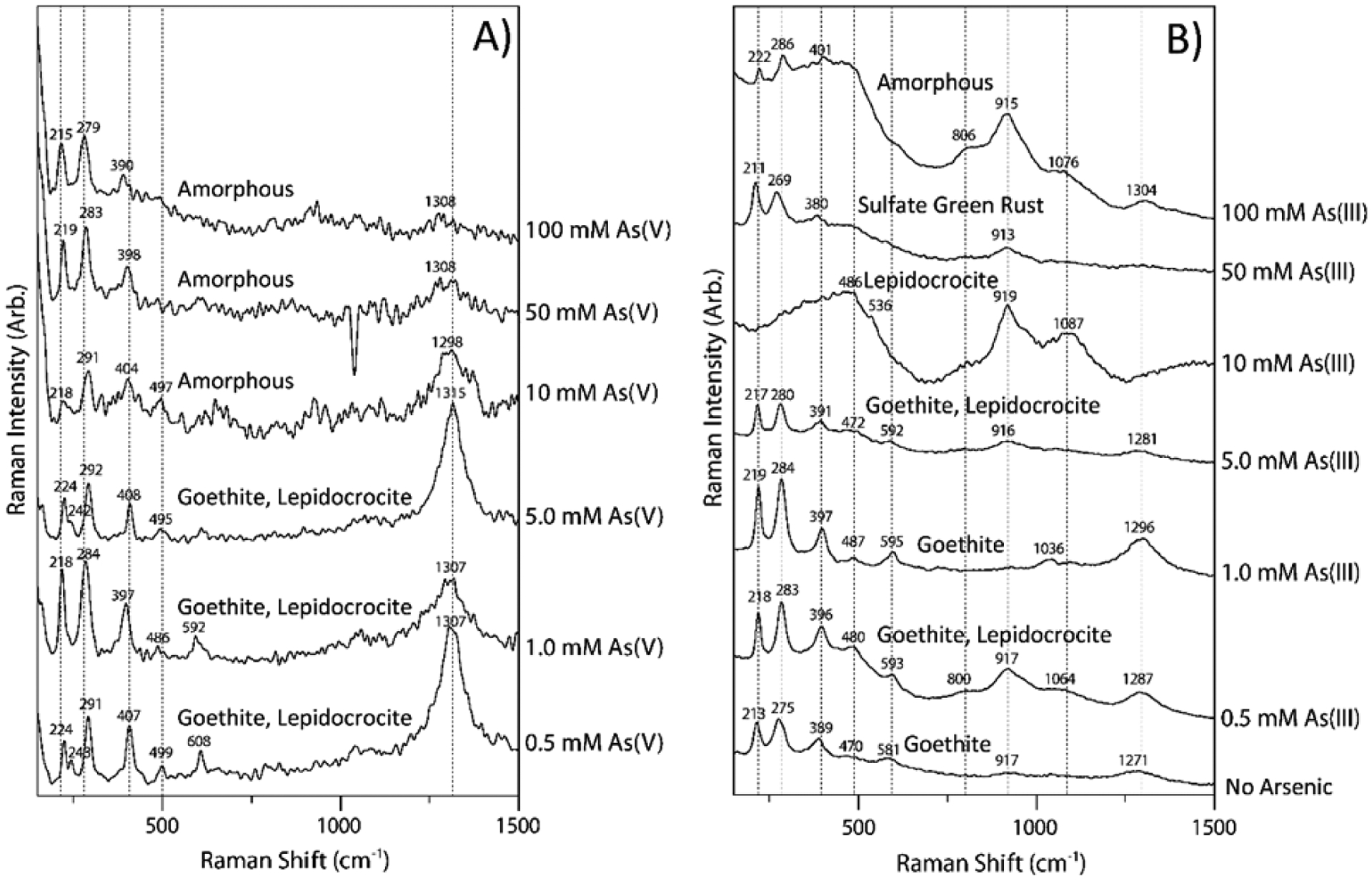
Raman spectra of coprecipitates formed at varying initial concentrations of dissolved As(V) (**A**) and As(III) (**B**).

Sulfate green rust formed only in the presence of Fe(II) and Fe(III) when As(III) was added at 50 mM (Figure 2), which can be described by Equation (2) (for simplicity, the solid Fe(II): Fe(III) ratio in the formed SGR is assumed to be unity):

(2) $${\text{Fe}}^{2+}\;+\;{\text{Fe}}^{3+}\;+\;3{\text{OH}}^{-}\;+\;{\text{SO}}_{\text{4}}{}^{2-}\;+\;{\text{nH}}_{2}\text{O}\;=\;\text{Fe}(\text{II})\text{Fe}(\text{III}){\left(\text{OH}\right)}_{3}{\text{SO}}_{4}\cdot {\text{nH}}_{2}\text{O}$$

At 100 mM As(III), amorphous HFO was formed (Figure 2). Lepidocrocite was identified when As(III) was added at 10 mM; goethite and lepidocrocite were formed at initial As(III) concentrations of 1.0 and 5.0 mM when the equilibrium pH values were 3.73 and 4.37 (Figure 2, Table 2). An earlier thermodynamic analysis showed that SGR is metastable vs. magnetite, except in a limited domain around pH 6–7.5 when ferrous and ferric sulfate is titrated with NaOH [56]. Later studies showed uncertainty in thermodynamic data on sulfate green rust [57,58]. Because the kinetics of mineral formation were not evaluated in the present work, SGR may exist under metastable conditions contrary to the stable phases magnetite and/or lepidocrocite.

### 3.3. Raman Spectra of Coprecipitates

Goethite formed in the absence of As showed Raman shifts at 213, 275, 389, 470, 581, and 1271 cm^−1^, which are close to the bands at 225, 297, 393, 482, and 565 cm^−1^ reported by Legodi and de Waal [59] for goethite. Raman shifts of the goethite formed in the presence of 1.0 mM As(III) were slightly increased to 219, 284, 397, 487, 595, and 1296 cm^−1^ (Figure 2B). Lepidocrocite formed in the presence of 10 mM As(III) had fewer Raman bands at 486, 536, 919, and 1087 cm^−1^. Sulfate green rust formed in the presence of 50 mM As(III) exhibited Raman bands at 211, 269, 380, and 913 cm^−1^. These bands are slightly different from the spectra of carbonate green rust [60] that exhibits two strong and sharp bands at 433 and 511 cm^−1^, several weak and sharp bands at 222, 260, 433, and 1057 cm^−1^, and two broad and weak bands at 157 and 670 cm^−1^. The two strong bands at 433 and 511 cm^−1^ are attributed to Fe^2+^–OH and Fe^3+^–OH stretchings, respectively [61]. Compared to sulfate green rust, the amorphous solids formed at the highest As(III) level of 100 mM showed higher Raman shifts. With the exception of sulfate green rust, the presence of either As(V) or As(III) resulted in slight increases in Raman shifts in major Raman bands of minerals as compared to goethite without As (Figure 2A,B). Arsenite may reside in the interlayers of sulfate green rust, as well as on the edges of the crystals, which may cause Raman band shifts.

### 3.4. Extent and Nature of Coprecipitated As(V) and As(III)

**Figure 3.**
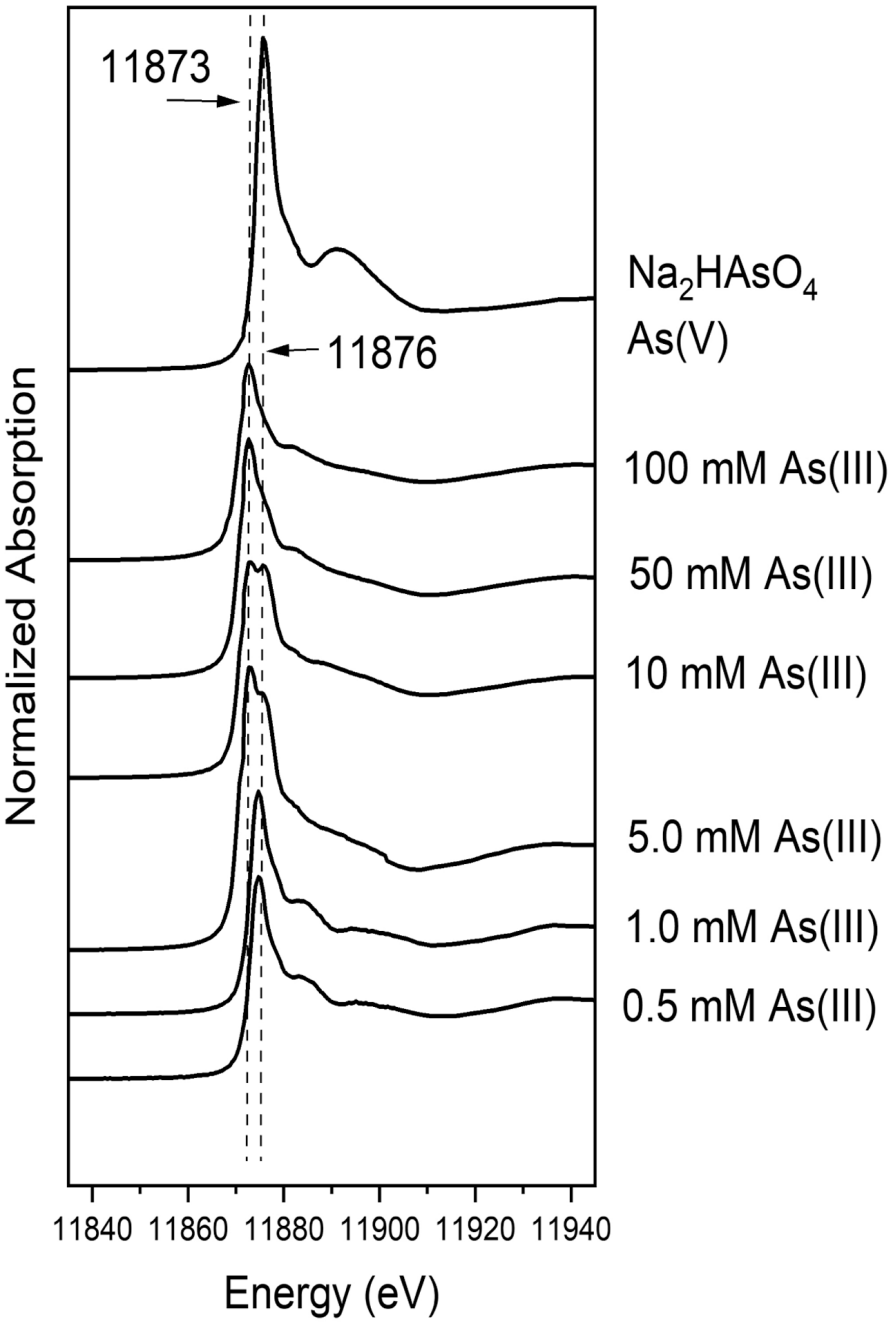
Normalized As XANES spectra and quantitative fit deconvolutions with reference As(V) (11876 eV; white line) and As(III) (11873 eV; white line) and spectra for coprecipitates at various initial As(III) concentrations (0.5–100 mM).

The As XANES spectra of coprecipitates are compared with reference sodium arsenate which presents as As(V), as indicated by the characteristic X-ray absorption maximum at 11876 eV (Figure 3). The vertical dashed line in Figure 3 is plotted at the peak position above the edge in the spectra of As(V) coprecipitates to facilitate the comparison of edge shift. The As XANES spectrum of the coprecipitated sample at the highest As(III) solution (100 mM) showed strong absorption maxima as As(III) 11,873 eV (absorption maxima 11,871–11,873 eV); however, small amounts of As(V) were expected to occur in the sample because of high post-edge slopes of As(III).

Co-precipitate at 50 mM As(III) showed strong absorption as As(III) with a slight hump which indicated As(V) (absorption maxima 11,875–11,876 eV). The maximum X-ray absorption of As(V) in each coprecipitate increased with lower initial As(III) concentration. Coprecipitates with As(III) solutions in batch experiments showed mixtures of As(III) and As(V). The relative proportions of As(III) and As(V) in solids were semi quantified by linear combination fits of the XANES spectra. We used As(III) and As(V) adsorbed to goethite for the fit end members.

Table 3 shows the percentage of As(III) oxidizedto As(V) from the linear combination of fits estimate. The amount of As(III) oxidation to As(V) observed by XANES in the coprecipitates was proportional to the total initial As(III) concentrations in solution. Normalized XANES spectra are sensitive to the proportions of components present but not to the absolute amount of each. Furthermore, elements in different oxidation states have different absorption properties such that the apparent proportion derived from fitting XANES spectra may not necessarily equal the component fraction quantitatively.

## 4. Discussion

### 4.1. Stabilization of Sulfate Green Rust by As(III)

Both As(V) and As(III) adsorption occurs primarily as bidentate binuclear (^2^*C*) inner-sphere surface complexes and the preferred adsorption sites are at sulfate green rust edges when green rust was formed before reacting with added As [62]. In our coprecipitated SGR, similar mechanisms may be operative for arsenic immobilization, but more detailed studies are needed to compare the pre-formed SGR and coprecipitated SGR. The presence of 50 mM As(III) facilitated the formation of SGR because the final equilibrium pH was high enough (pH > 6.3) and because As(III) may have entered the interlayer space replacing some sulfate ions in the SGR structure. A recent study showed that the presence of As(III) at 200 mM limits the polymerization of Fe(II) at pH 7 and the formation of GR, and inhibits the formation of goethite and lepidocrocite [40]. No SGR was formed in the As(V) system. At As(V) concentrations from 0.5 to 50 mM, the pH values ranged from 4.83 to 6.23, insufficient to induce SGR precipitation. X-ray amorphous solids were formed at As(V) concentrations of 10, 50, and 100 mM (Figure 1A). Amorphous ferric arsenate sulfate has been reported to form in acid mine drainage [63]. It is possible that it also formed in this study.

### 4.2. Was Dissolved O_2_ An Oxidant for As(III) Oxidation?

Dissolved Fe(II) concentrations at 24 h after coprecipitation were still two orders of magnitude higher than the initial dissolved O_2_ in the centrifuge tubes (Tables 1 and 2). The solubility of O_2_ at 21 °C is about 9 mg L^−1^, corresponding to 0.28 mM dissolved O_2_, much less than the initial concentration of 133 mM Fe(II) and the equilibrium concentrations of Fe(II) (27.8–98.1 mM). Because the oxidation reaction of dissolved Fe(II) by dissolved O_2_ is fast (on the order of minutes), the dissolved O_2_ should have been consumed quickly by dissolved Fe(II). On the other hand, oxidation of dissolved As(III) by air is much slower with a half-life of 4–9 days [64]. Nevertheless, previous studies [65] show that the oxidation of As(III) can occur under oxic conditions through Fenton reactions involving reactive oxygen species (e.g., O_2_^−^, H_2_O_2_, or OH) formed as intermediate species during the oxidation of Fe(II) by dissolved O_2_. The Fenton oxidation of As(III) has been shown to be catalyzed by ferrihydrite [66,67]. We show here that goethite and lepidocrocite possess a similar catalytic effect. Previously we have shown that carbonate GR in the absence of O_2_ also partially oxidized As(III) in aqueous solution. That experiment was conducted in an anaerobic glovebox however it might be extremely difficult to maintain a completely anoxic condition. Nevertheless, other studies from different research groups have demonstrated that goethite and schwertmannnite partially oxidize sorbed As(III) to As(V), even in anoxic media at pH 3 [68]. The presence of O_2_ may be a sufficient but not essential condition for As(III) oxidation to take place. Other species such as Fe(IV), rather than the hydroxyl radical, were shown to be the oxidant for As(III) oxidation [69]. Future studies should be carried out in the absence of dissolved O_2_ to elucidate the detailed mechanism of As(III) oxidation by dissolved O_2_ as compared to dissolved Fe(III) and structurally bound Fe(III).

Previous studies showed that As(III) is partly oxidized by reactive intermediates (possibly an Fe(IV) species) formed during the oxidation of Fe(II) by O_2_ [65,70,71]. Our earlier study showed that preformed carbonate green rust itself acts as an oxidizer for As(III) even in the absence of dissolved O_2_ [36]. In addition to green rust, iron oxides such as goethite and lepidocrocite may also be involved in oxidizing As(III) to As(V) (Table 2) as shown in Equation (3).

(3) $$2\alpha (\text{or}\;\gamma )\;-\;\text{FeOOH}\;+\;{\text{H}}_{2}{\text{AsO}}_{\text{3}}{}^{-}\;+\;3{\text{H}}^{+}\;=\;2{\text{Fe}}^{2+}\;+\;{\text{HAsO}}_{\text{4}}{}^{2-}\;+\;2{\text{H}}_{2}\text{O}$$

Oxidation of As(III) also occurred in SGR system as shown in Equation (4).

(4) $$2\text{Fe}(\text{II})\text{Fe}(\text{III}){\left(\text{OH}\right)}_{3}{\text{SO}}_{4}\cdot {\text{nH}}_{2}\text{O}\;+\;3{\text{H}}^{+}\;+\;{\text{H}}_{2}{\text{AsO}}_{\text{3}}{}^{-}\;=\;4{\text{Fe}}^{2+}\;+\;{\text{HAsO}}_{\text{4}}{}^{2-}\;+\;2{\text{SO}}_{\text{4}}{}^{2-}\;+\;(\text{n}\;+\;5){\text{H}}_{2}\text{O}$$

Recent X-ray absorption spectroscopic evidence showed that As(V) sorbs to green rust and magnetite by forming bidentate inner-sphere surface complexes, resulting from corner sharing between AsO_4_ groups and FeO_6_ octahedra [11]. As(III) also forms inner-sphere surface complexes on green rust and magnetite [11]. The interaction of aqueous As(III) with magnetite during its precipitation from aqueous solution at neutral pH includes surface adsorption and surface precipitation reactions, which in turn influence the crystal growth of magnetite [72]. EXAFS spectroscopy studies showed that As(III) forms predominantly tridentate hexanuclear As(III)O_3_ complexes (^3^*C*), where the As(III)O_3_ pyramids occupy vacant tetrahedral sites on {111} surfaces of magnetite particles [72]. In addition, As(III) tends to form mononuclear edge-sharing As(III)O_3_ species (^2^*E*) within a highly soluble amorphous As(III)-Fe(II, III)-containing precipitate [11].

### 4.3. Implications of Iron-Based Remediation of Groundwater

Zerovalent iron (ZVI) has been used to treat simulated groundwater [27,73] and real groundwater at a contaminated site [74]. Iron corrosion products such as iron oxides and green rusts are major scavengers for both As(V) and As(III) in ZVI-based groundwater remediation [27,75]. Sorbed As may undergo further biogeochemical redox transformation. Further studies should evaluate the fate of the sorbed As as a function of time as ZVI and ZVI corrosion products age in the subsurface, as permanent sequestration of As is desired.

## 5. Conclusions

In summary, this research showed that SGR forms in the presence of As(III) at a pH near neutral (pH 6.3); whereas, iron oxides (goethite and lepidocrocite) form at pH 4.1–5.8 and amorphous ironand As-containing solids form at very high initial As(III) concentrations (100 mM) or moderate to very high As(V) concentrations (10–100 mM). Factors such as pH and structural stabilizers (surface complexing anions) are determinants in the formation of green rusts. As(III) is partially oxidized to As(V) in the coprecipitation process. Regardless of the different solid phases formed (GRs and iron oxides), there is substantial removal of As(V) and As(III) by coprecipitation with them. Coprecipitation is advantageous because it removes more arsenic than adsorption of As by preformed HFO solids [76]. The presence of Fe(II) sorbed to surface sites of amorphous ferric hydroxide increases the capacity for As(V) sorption [77]. This study suggests that green rust and iron oxides formed in the presence of arsenic may remove large amounts of arsenic from groundwater when engineered systems (iron-based PRB) and natural attenuation approaches are used to remediate arsenic-laden groundwater. The fate and transformation of coprecipitated As over long term merits further investigation.

## Figures and Tables

**Table 1.** Coprecipitation of As(V) with Fe(II) and Fe(III) in sulfate solutions (initial [Fe]_0_ = 266 mM, nd = non-detect, HFO = hydrous ferric oxide).

[As(V)]_0_/mM	pH	Eh/mV	[Fe^2+^]/mM	[Fe]/mM	Fe Removal/%	[As(V)]/mM	[As(III)]/mM	As Removal/%	As conc. in solids/mg kg^−1^	Mineralogy	Solids Color	Magnetic
100	6.59	30.8	21.9	27.8	89.55	0.053	nd	99.95	171,000 ± 6000	amorphous HFO	gray	no
50	6.23	53.7	69.6	62.3	76.58	0.018	nd	99.96	101,000 ± 500	amorphous HFO	brown	no
10	6.15	61.6	56	84	68.42	nd	nd	100	40,600 ± 6300	amorphous HFO	brown	no
5	5.78	86.3	69.8	91	65.79	nd	nd	100	17,400 ± 800	lepido., goethite	brown	no
1	5	187	96.2	95.5	64.1	nd	nd	100	3980 ± 230	lepido., goethite	brown	no
0.5	4.83	227	86.2	95.1	64.25	nd	nd	100	2070 ± 100	goethite, lepido.	brown	no
0	4.09	366	97.1	96.7	63.65	nd	nd	nd	nd	goethite	brown	no

**Table 2.** Coprecipitation and oxidation of As(III) with Fe(II) and Fe(III) in sulfate solutions (initial [Fe]_0_ = 266 mM, nd = non-detect, HFO = hydrous ferric oxide).

[As(V)]_0_/mM	pH	Eh/mV	[Fe^2+^]/mM	[Fe]/mM	Fe Removal/%	[As(V)]/mM	[As(III)]/mM	As Removal/%	As conc. in solids/mg kg^−1^	Mineralogy	Solids Color	Magnetic
100	6.62	18.1	55.1	56.8	78.65	1.06	0.29	98.65	207,000 ± 7000	amorphous HFO	gray	no
50	6.34	56.8	47.9	75.6	71.58	0.22	0.32	98.92	136,300 ± 26,100	SGR	green	no
10	5.51	194	95.3	93.7	64.77	0.0039	0.071	99.25	31,600 ± 1150	lepidocrocite	brown	no
5	4.37	342	88.3	96.9	63.57	0.0024	0.047	99.01	14,900 ± 1700	goethite, lepido.	brown	no
1	3.73	417	92.2	98.1	63.12	0.00051	0.0070	99.25	3280±520	goethite, lepido.	brown	no
0.5	4.96	214	89.2	94	64.66	nd	0.0016	99.68	1850 ± 70	goethite	brown	no

**Table 3.** Arsenic XANES fits and estimated fractions of coprecipitated As(III) oxidized to As(V) from XANES fits.

Coprecipitate Sample	Component	Fitted XANES Fraction
100 mM As(III)	As(V)	0.110
	As(III)	0.890
50 mM As(III)	As(V)	0.131
	As(III)	0.869
10 mM As(III)	As(V)	0.294
	As(III)	0.706
5.0 mM As(III)	As(V)	0.509
	As(III)	0.491
1.0 mM As(III)	As(V)	0.722
	As(III)	0.278
0.5 mM As(III)	As(V)	0.722
	As(III)	0.278
